# Prevalence and Risk Factors for Hepatic Steatosis in Children With Perinatal HIV on Early Antiretroviral Therapy Compared to HIV-Exposed Uninfected and HIV-Unexposed Children

**DOI:** 10.3389/fped.2022.893579

**Published:** 2022-06-09

**Authors:** Penelope C. Rose, Etienne D. Nel, Mark F. Cotton, Richard D. Pitcher, Kennedy Otwombe, Sara H. Browne, Steve Innes

**Affiliations:** ^1^Department of Paediatrics and Child Health, Tygerberg Hospital and Stellenbosch University, Cape Town, South Africa; ^2^Family Center for Research With Ubuntu (FAMCRU), Cape Town, South Africa; ^3^Division of Radiodiagnosis, Department of Medical Imaging and Clinical Oncology, Faculty of Medicine and Health Sciences, Stellenbosch University, Cape Town, South Africa; ^4^Perinatal HIV Research Unit, Chris Hani Baragwanath Academic Hospital, Faculty of Health Sciences, University of the Witwatersrand, Johannesburg, South Africa; ^5^School of Public Health, Faculty of Health Sciences, University of the Witwatersrand, Johannesburg, South Africa; ^6^Department of Medicine, University of California, San Diego, San Diego, CA, United States; ^7^Desmond Tutu HIV Centre, University of Cape Town, Cape Town, South Africa

**Keywords:** paediatric, NAFLD, liver, fatty liver, hepatic fibrosis

## Abstract

**Objectives:**

We evaluated the prevalence and risk factors for hepatic steatosis in South African children with perinatally acquired HIV (PHIV) who started treatment early and remain on long-term antiretroviral therapy (ART) compared to HIV-uninfected children.

**Design:**

A cross-sectional study from April 2019 to October 2021. PHIV, HIV-exposed uninfected (HEU) and HIV-unexposed (HU) children were enrolled from an ongoing cohort study.

**Methods:**

All children had transient elastography (TE) with controlled attenuation parameter (CAP). Liver enzymes, lipogram, insulin and glucose were sent after an overnight fast. Multivariable linear regression analyses identified predictors of CAP. Hepatic steatosis was defined as CAP>248kPa.

**Results:**

215 children (111 [52%] male; median age 14.1 years; IQR 12.7–14.9) participated in the study, 110 PHIV, 105 HIV-uninfected (36 HEU, 69 HU). PHIV initiated ART at a median age of 2.7 months (IQR 1.8–8.5). Hepatic steatosis prevalence was 9% in PHIV, 3% in HEU and 1% in HU children (*p* = 0.08). However, 8% of lean (body mass index *z*-score ≤ +1) PHIV had hepatic steatosis compared to zero lean HEU or HU children (*p* = 0.03). In multivariable linear regression analysis of all PHIV, body mass index (BMI) z-score was positively associated with CAP (*p* = 0.001) while CD4 count (*p* = 0.02) and duration of suppression of HIV viraemia (*p* = 0.009) were negatively associated with CAP, adjusting for age, sex and ethnicity.

**Conclusions:**

Hepatic steatosis prevalence was higher in lean PHIV than lean HIV-uninfected South African children. Longer suppression of HIV viraemia and higher CD4 count were associated with lower CAP and might be protective factors for hepatic steatosis in PHIV children.

## Introduction

Despite a substantial reduction in HIV-associated morbidity and mortality with antiretroviral therapy (ART), liver disease remains a leading cause of non-AIDS death in adults with HIV (1–3). In addition to hepatitis B and C, which account for a large proportion of this burden, non-alcoholic fatty liver disease (NAFLD) is increasingly recognised as a cause of liver disease in adults with HIV (4–6). NAFLD includes a spectrum of disease from simple hepatic steatosis to the inflammatory non-alcoholic steatohepatitis (NASH), and may lead to hepatic fibrosis and cirrhosis. NAFLD is more common among adults with HIV than the general population, occurring at a lower body mass index and with higher rates of progression to NASH and fibrosis (3, 7–10).

The presence and severity of hepatic fibrosis is of important prognostic significance in children with chronic liver diseases, including NAFLD. The current reference standard to diagnose fibrosis, liver biopsy, is invasive and entails a risk of serious complications. Various non-invasive markers have been developed to evaluate hepatic fibrosis in adults, including the AST-to-platelet ratio index (APRI) and Fibrosis-4 (FIB-4) index, which have been used in children (11, 12).

Transient elastography (TE) is a non-invasive ultrasound-based technology using low-frequency elastic waves to measure liver stiffness as a surrogate for liver fibrosis (13–16). Controlled attenuation parameter (CAP) can be measured simultaneously to quantify hepatic steatosis based on ultrasound signal attenuation (17). TE has performed well in identifying children with significant liver fibrosis and is the method of choice to monitor the longitudinal progression of liver fibrosis in children (13, 14, 18, 19). CAP is useful to diagnose hepatic steatosis in adults and has also been used in children (17, 20–22).

Although NAFLD is increasing in adults and children worldwide with rising obesity, little is known about HIV-associated NAFLD in low- and middle-income countries (LMICs) including Sub-Saharan Africa, the region with the largest global burden of HIV. To our best knowledge there is currently no published literature on NAFLD in children living with perinatally acquired HIV (PHIV) in Africa. Our Center cares for children living with PHIV who participated in early treatment clinical trials [median age at ART initiation months 2.7 (1.8–8.5)]. The aim of this study was to describe the prevalence and risk factors for hepatic steatosis in early treated PHIV children on long-term ART, compared to HIV-uninfected (HU and HEU) controls of similar age, race/ethnicity and socioeconomic background, using non-invasive hepatic investigations.

## Materials and Methods

### Study Design, Setting and Participants

In this cross-sectional study from April 2019 to October 2021, children were consecutively enrolled from an existing cohort of PHIV and HIV-uninfected controls at the Family Centre for Research with Ubuntu (FAMCRU), Tygerberg Hospital, Cape Town, South Africa. Recruitment was interrupted from March to October 2020 due to the Covid-19 pandemic and national lockdown. The cohort comprised PHIV on ART recruited from local communities after birth or during the first years of life. These children had participated in either the Children with HIV Early antiRetroviral (CHER) trial or the P1060 trial (23, 24). The CHER trial was an ART strategy trial where PHIV were randomised to either early time-limited or deferred continuous ART from early infancy. P1060, conducted through the International Maternal Paediatric Adolescent AIDS Clinical trial (IMPAACT) group, compared nevirapine to lopinavir-ritonavir in ART-naïve children who all received zidovudine and lamivudine nucleoside reverse transcriptase inhibitors (23, 24). Children without HIV were either perinatally HIV-exposed but uninfected (HEU) or perinatally HIV-unexposed and HIV-uninfected (HU) and came from a vaccine study linked to the CHER trial or from a neurocognitive substudy following P1060. Children were followed for non-communicable diseases (NCDs) at FAMCRU (R01HD083042). No exclusion criteria were applied and children were only excluded from the analysis if without a reliable transient elastography result. Individual participant clinical assessments, blood sample collections and transient elastography were performed on the same day. Ethical approval was obtained from the Stellenbosch University Health Research Ethics Committee (N12/11/076 and S20/02/046[PhD]). Written informed consent was obtained from a parent or legal guardian and written assent from all child participants.

### Clinical Variables

Demographic and clinical data were collected both by structured interview and medical record review, including age, gender, ethnicity, Tanner staging of puberty, current medical conditions, current and past medication, herbal or alternative remedies, alcohol consumption and illicit substance usage. For PHIV children, past and present ART, duration of suppressed HIV viraemia, and most recent CD4 T-cell count during the past year were documented from clinical records. Anthropometric data included weight measured to the nearest 0.1 kg in light clothing and height using a stadiometer, waist circumference at the umbilicus and hip circumference measured around the buttocks at the largest circumference, all measured to the nearest 0.1 centimetres. Body mass index (BMI) z-scores were calculated using World Health Organization AnthroPlus software (www.who.int/growthref/tools/en/). Children with a BMI z-score below +1 were classified as lean, between +1 and +2 as overweight and over +2 as obese, respectively (25). Children were classified as being either prepubertal (Tanner stage 1) or pubertal (Tanner stage ≥ 2).

Blood tests for glucose, insulin, platelets, triglycerides (TG), cholesterol [total, high-density lipoprotein (HDL) and low-density lipoprotein (LDL)], alanine transaminase (ALT), aspartate transaminase (AST) and HIV RNA PCR viral load in PHIV children were obtained after an overnight fast. All specimens were analysed at the National Health Laboratory Service (NHLS) at Tygerberg Hospital. The homeostatic model assessment for insulin resistance score (HOMA) was calculated as fasting insulin (mIU/mL) x fasting glucose (mmol/L)/22.5. If liver stiffness was >7kPa or CAP was ≥238 dB/m or if the transaminases were elevated more than twice the upper limit of normal, standard clinical tests were performed to exclude viral hepatitis, autoimmune hepatitis, Wilson's disease and alpha-1-antitrypsin deficiency. These included hepatitis B surface antigen, hepatitis C antibody, auto-antibodies (anti-nuclear, anti-smooth muscle and anti-liver kidney), total IgG, caeruloplasmin and alpha-1-antitrypsin level. APRI was calculated as AST x 100/[platelet count (10^9^/L) x AST upper limit of normal]. FIB-4 index was calculated as age (years) x AST (U/L)/[platelets (10^9^/L) x ALT^1/2^ (U/L)]. A cut-off of APRI >0.5 and FIB-4 >1.45 were defined as being suggestive of hepatic fibrosis (26). TG-to-HDL ratio was used as a surrogate marker for small dense atherogenic LDL (27, 28). The triglyceride-glucose index (TyG) was calculated as a marker of insulin resistance as ln [fasting TG (mg/dL) x fasting glucose (mg/dL)].

### Liver Ultrasound

Sonographic examination of the liver was performed on children enrolled before the Covid-19 pandemic and lockdown from April 2019 to February 2020, but not post-lockdown. Ultrasounds were performed by a trained experienced ultrasonographer using a Toshiba Aplio 400 ultrasound machine and 3.75 MHz curvilinear probe preset to standard abdominal settings. All abdominal ultrasounds were reported by an experienced senior radiologist (RDP) blinded to all clinical details. Hepatic steatosis was graded as 0 (normal) with normal liver echogenicity, 1 (mild steatosis) a diffuse increase in liver echogenicity with normal visualisation of intrahepatic vessels and diaphragm, 2 (moderate steatosis) increased liver echogenicity with impaired visualisation of vessel walls and the diaphragm and 3 (severe steatosis) was non-visualisation of the hepatic vessels and diaphragm due to markedly increased echogenicity (29–31).

### Transient Elastography and Controlled Attenuation Parameter

All children had liver stiffness and CAP measured using the Fibroscan^®^ (Echosens: Paris, France) with the M probe after an overnight fast. All measurements were performed by one of two experienced operators. A scan was considered reliable if at least 10 valid shots were obtained (>60% successful and interquartile range <30% of the median) (32). A CAP cut-off of >248dB/m, which has previously been described as highly specific for diagnosing liver steatosis in children and was used in studies of hepatic steatosis in young adults living with HIV, was used to classify children as having hepatic steatosis (22, 33).

### Statistical Methods

Results were expressed as frequencies and percentages for categorical variables, and medians and interquartile ranges (IQR) for continuous variables. Comparisons of categorical variables used either the Chi-square or Fisher's exact test as appropriate. To compare continuous variables of different groups with three or more categories, one-way analysis of variance was used for normally distributed variables with equal variances or the Kruskal-Wallis test if these assumptions were not met. Coefficients of variation were calculated as (IQR/median) x 100 for non-normally distributed continuous variables. Scatter diagrams were used to plot CAP against anthropometric, metabolic and HIV-specific risk factors. Correlations between CAP and anthropometric and metabolic risk factors were calculated using Spearman's correlation coefficient. Dot plots were used to compare CAP between PHIV, HEU and HU children in lean and overweight or obese children. The kappa statistic was calculated to measure the agreement between CAP and ultrasound to diagnose hepatic steatosis.

Univariable linear regression analyses were performed to identify risk factors associated with CAP. CAP was log transformed to approximate a normal distribution for the linear regression analysis and reverse transformed for interpretation. All variables with a *p*-value < 0.1 were evaluated for inclusion in multiple linear regression analyses to identify risk factors associated with CAP. Age, sex and ethnicity were retained in the final multivariable models as possible confounders. Variance inflation factors (VIF) were calculated for independent variables to detect multicollinearity. All analyses were two-tailed, *p* < 0.05 was considered statistically significant and used STATA version 12 (StataCorp LP, College Station, Texas, USA).

## Results

During the study period, 225 children were evaluated. 215 (96%) children with a reliable transient elastography result were included in the study, 110 (51%) PHIV and 105 (49%) HIV-uninfected, 36 HEU and 69 HU. All but 3 PHIV had undetectable viral loads (<50 HIV-1 RNA copies/ml). These three children had previously been virologically suppressed since early life. The majority of PHIV and HEU children were of African ethnicity. PHIV had a higher waist-hip ratio, higher triglycerides, higher triglyceride-to-HDL ratio and higher TyG index than HEU and HU children. HEU children had a lower total and LDL cholesterol than PHIV and HU children and lower triglycerides, TG-to-HDL ratio and TyG than PHIV. Characteristics comparing PHIV, HEU and HU children are provided in Table 1.

**Table 1:** Patient characteristics by HIV infection and exposure status.

	**PHIV** ***n*** **= 110**	**HEU** ***n*** **= 36**	**HU** ***n*** **= 69**	***P*****-value** **Overall**	***P*****-value** **PHIV vs. HEU**	***P*****-value** **HEU vs. HU**
**Demographics and clinical characteristics**						
Median age in years (IQR)	14.3 (13.1–14.8)	13.9 (12.5–15.5)	13.4 (12.0–14.7)	0.2	0.6	0.4
Sex, male	53 (48%)	20 (56%)	38 (55%)	0.6	0.4	1.0
Tanner staging						
Prepubertal (stage 1)	7 (6%)	5 (14%)	5 (7%)	0.3	0.2	0.3
Pubertal (stage ≥2)	103 (93%)	31 (86%)	64 (93%)			
Ethnicity						
African	99 (90%)	31 (86%)	31 (45%)	<0.0001	0.5	<0.0001
Mixed ethnicity	11 (10%)	5 (14%)	38 (55%)			
Median anthropometric parameters [Median (IQR)]						
BMI z-score	−0.02 (−0.81–0.66)	−0.15 (−0.76–0.24)	0.01 (−0.72–0.87)	0.6	0.8	0.5
Waist circumference (cm)	66.5 (62.3–72.7)	64.8 (61.9–67.9)	69.7 (63.4–74.9)	0.1	0.3	0.06
Waist-hip ratio	0.83 (0.79–0.86)	0.79 (0.77–0.84)	0.81 (0.78–0.84)	0.01	0.02	0.1
Weight category						
Lean (BMI z-score ≤ +1)	93 (85%)	33 (92%)	55 (80%)	0.5	0.5	0.3
Overweight (BMI z-score > +1 to +2)	11 (10%)	1 (3%)	8 (11%)			
Obese (BMI z-score >+2)	6 (5%)	2 (6%)	6 (9%)			
**Fasting metabolic profiles [Median (IQR)]**						
Total cholesterol (mmol/L)	4.10 (3.64–4.69)	3.60 (3.03–4.03)	3.97 (3.49–4.35)	0.001	0.0003	0.006
TG (mmol/L)	0.90 (0.69–1.29)	0.57 (0.40–0.79)	0.63 (0.49–0.75)	0.0001	<0.0001	0.4
LDL (mmol/L)	2.23 (1.77–2.76)	1.43 (1.38–2.34)	2.25 (1.82–2.62)	0.02	0.005	0.005
HDL (mmol/L)	1.37 (1.20–1.59)	1.44 (1.21–1.62)	1.38 (1.19–1.60)	0.9	0.9	1.0
TG-to-HDL ratio	0.65 (0.45–0.96)	0.38 (0.30–0.54)	0.45 (0.35–0.65)	0.0001	<0.0001	0.3
Glucose (mmol/L)	4.5 (4.3–4.9)	4.6 (4.1–4.9)	4.6 (4.4–4.8)	0.9	1.0	0.6
Insulin (μIU/mL)	10.7 (7.7–17.5)	8.6 (5.7–14.6)	9.0 (6.4–14.3)	0.05	0.07	1.0
HOMA	2.20 (1.50–3.38)	1.65 (1.24–3.05)	1.80 (1.21–3.07)	0.08	0.09	0.8
TyG	7.9 (7.6–8.3)	7.5 (7.1–7.8)	7.6 (7.3–7.8)	0.0001	<0.0001	0.4
**Immune and viral suppression [Median (IQR)]**						
CD4 (cells/uL) at ART start	1327 (756–1849)	-	-			
CD4% at ART start	26.2 (19.0–36.7)	-	-			
Most recent CD4 (cells/uL)	818 (654–1011)	-	-			
% with suppressed HIV viral load (<50 copies/ml)	97%	-	-			
Duration of suppression of HIV viraemia (years)	9.2 (4.6–12.1)	-	-			
Median age at ART initiation (months)	2.7 (1.8–8.5)	-	-			
Initial antiretrovirals		-	-			
Zidovudine	110 (100%)					
Lamivudine	110 (100%)					
Lopinavir/ritonavir	97 (88%)					
Nevirapine	13 (12%)					
Current antiretrovirals						
Abacavir	23 (21%)					
Zidovudine	76 (69%)					
Tenofovir	10 (9%)					
Lamivudine	107 (97%)					
Emtricitabine	1 (1%)					
Efavirenz	10 (9%)					
Nevirapine	7 (6%)					
Lopinavir/ritonavir	77 (70%)					
Atazanavir/ritonavir	4 (4%)					
Darunavir	2 (2%)					
Dolutegravir	11 (10%)					

*ALT, alanine transaminase; APRI, AST-to-platelet ratio index; ART, antiretroviral therapy; AST, aspartate transaminase; BMI, body mass index; CAP, controlled attenuation parameter; HDL, high-density lipoprotein; HEU, HIV-exposed uninfected; HOMA, homeostatic model assessment; HU, HIV-unexposed; LDL, low-density lipoprotein; PHIV, children with perinatally acquired HIV; TE, transient elastography; TG, triglycerides; TyG, triglyceride-glucose index*.

### Concurrent Conditions and Medications

No participants were diagnosed with any medical condition known to cause hepatic steatosis or fibrosis, other than obesity and HIV. PHIV children had significantly more concurrent conditions (*p* < 0.0001), 62 (56%) compared to only 4 (11%) HEU and 9 (13%) HU children. Concurrent conditions in PHIV children were primarily allergic conditions (asthma, allergic rhinitis and eczema) in 37 (34%) PHIV, 1 (3%) HEU and 5 (7%) HU children and neurodevelopmental delay with/without attention deficit hyperactivity disorder (ADHD) seen in 34 (31%) PHIV, 1 (3%) and 3 (4%) HU children. Of these 40 (36%) PHIV, 2 (6%) HEU and 7 (10%) HU children were concurrently receiving medication for these concurrent conditions; methylphenidate for ADHD and antihistamines and/or low to medium dose topical corticosteroids for allergic conditions. None of the PHIV children who were not receiving treatment reported symptoms requiring additional treatment at the time of assessment. No children reported using herbal or alternative medications. One PHIV child was being treated for drug-susceptible tuberculosis, one PHIV child was receiving isoniazid prophylaxis and another PHIV had completed a course of isoniazid prophylaxis a month prior to evaluation. All PHIV children in this post-trial cohort received zidovudine as part of their ART regimen, the majority still receiving zidovudine when assessed for this study. No children received stavudine and 2 PHIV children previously received didanosine, neither of whom had hepatic steatosis. No children tested positive for hepatitis B or C infection. All children were clinically well at the time of assessment, with no evidence of any opportunistic infections. No children reported alcohol consumption above the upper safe consumption levels for adults and only 1 HU child reported a single episode of alcohol consumption during the past month.

### Liver Enzymes and Non-invasive Hepatic Fibrosis Scores

ALT was higher in PHIV than HEU or HU children (Table 2). Six (6%) boys (5 PHIV and 1 HEU) and 6 (6%) girls (all PHIV) had mildly raised ALT above the upper limit of normal for age and sex (22U/l for girls, 26U/l for boys), with no boys and 2 PHIV girls having an ALT more than twice the upper limit of normal. Two PHIV children had both a raised ALT and liver stiffness >7.0 kPa. Two children, one PHIV and one HEU, had raised ALT and CAP >248 dB/m. Abnormal APRI was present in 2 (2%) PHIV and no HEU or HU. One PHIV child with an APRI of 0.53 had a raised liver stiffness of 7.4kPa; the other PHIV child had an APRI of 0.94 and normal liver stiffness of 4.6kPa. Abnormalities in Fib-4 were not found.

**Table 2:** Liver ultrasound, enzymes, transient elastography and controlled attenuation parameter results.

	**PHIV *n* = 110**	**HEU** ***n*** **= 36**	**HU *n* = 69**	***P*****-value** **overall**	***P*****-value** **PHIV vs. HEU**	***P*****-value** **HEU vs. HU**
**TE and CAP [Median (IQR)]**
Liver stiffness (kPa)	5.0 (4.4–5.9)	5.1 (4.6–6.1)	5.1 (4.5–5.8)	0.4	0.2	0.2
Liver fibrosis (>7kPa)	8 (7%)	2 (6%)	1 (1%)	0.2	1.0	0.3
CAP (dB/m)	195 (173–223)	171 (157–197)	186 (160–212)	0.01	0.004	0.3
Hepatic steatosis (>248dB/m)	10 (9%)	1 (3%)	1 (1%)	0.08	0.3	0.6
CAP (dB/m) in lean children	190 (173–223)	171 (158–195)	182 (157–203)	0.003	0.2	0.3
**Liver enzymes and biomarkers [Median (IQR)]**
ALT (U/L)	15 (12–20)	13 (10–15)	12 (10–15)	0.0001	0.002	0.3
Raised ALT	11 (10%)	1 (3%)	0 (0%)	0.008	0.3	0.3
AST (U/L)	24 (21–29)	22 (19–27)	22 (18–24)	0.07	0.3	0.2
APRI	0.20 (0.16–0.23)	0.19 (0.15–0.22)	0.17 (0.15–0.23)	0.8	0.7	0.8-
Raised APRI	2 (3%)	0 (0%)	0 (0%)	0.7	1.0	0.4
FIB-4	0.07 (0.05–0.08)	0.07 (0.06–0.09)	0.08 (0.06–0.10)	0.3	0.2	
Liver sonar–hepatic steatosis grade	*n* = 31	*n* = 15	*n* = 34			
0	11 (36%)	7 (47%)	25 (74%)	0.009	0.2	0.09
1	19 (61%)	6 (40%)	8 (24%)			
2	1 (3%)	2 (13%)	1 (3%)			
3	0 (0%)	0 (0%)	0 (0%)			

### Liver Ultrasound

Eighty (37%) children had a liver ultrasound, 31 (28%) PHIV, 15 (42%) HEU and 34 (49%) HU children. No children had severe (grade 3) hepatic steatosis, but 20 (65%) PHIV had evidence of either mild (grade 1) or moderate (grade 2) steatosis on liver ultrasound, compared to 8 (53%) HEU and 9 (26%) HUU children (Table 2). Median CAP was 270 dB/m (IQR 230-298 dB/m) in children with grade 2 steatosis on ultrasound, significantly higher than in those with grade 1 (median CAP 197 dB/m, IQR 160-218 dB/m; ANOVA *p* = 0.008) and grade 0 (median 195 dB/m, IQR 167–227 dB/m; ANOVA *p* = 0.004) steatosis, respectively.

### Transient Elastography

Median liver stiffness did not differ by HIV infection or exposure status (Table 2). Liver stiffness was significantly correlated with age (Spearman's rho 0.21; *p* = 0.002) and APRI (Spearman's rho = 0.30; *p* = 0.0002), but not FIB4, CAP, BMI z-score, waist circumference or ALT level. The coefficient of variation for liver stiffness was 28%. There were 8 (7%) PHIV, 2 (6%) HEU and 1 (1%) HU children with an elevated liver stiffness >7.0 kPa. Only one of these children, a lean PHIV girl, had both a raised liver stiffness and raised CAP, but normal liver enzymes. Three children (1%), one each of PHIV (1%), HEU (3%) and HU (1%) had liver stiffness >9 kPa, which has been associated with advanced hepatic fibrosis in children.

### Controlled Attenuation Parameter

**Figure 1:**
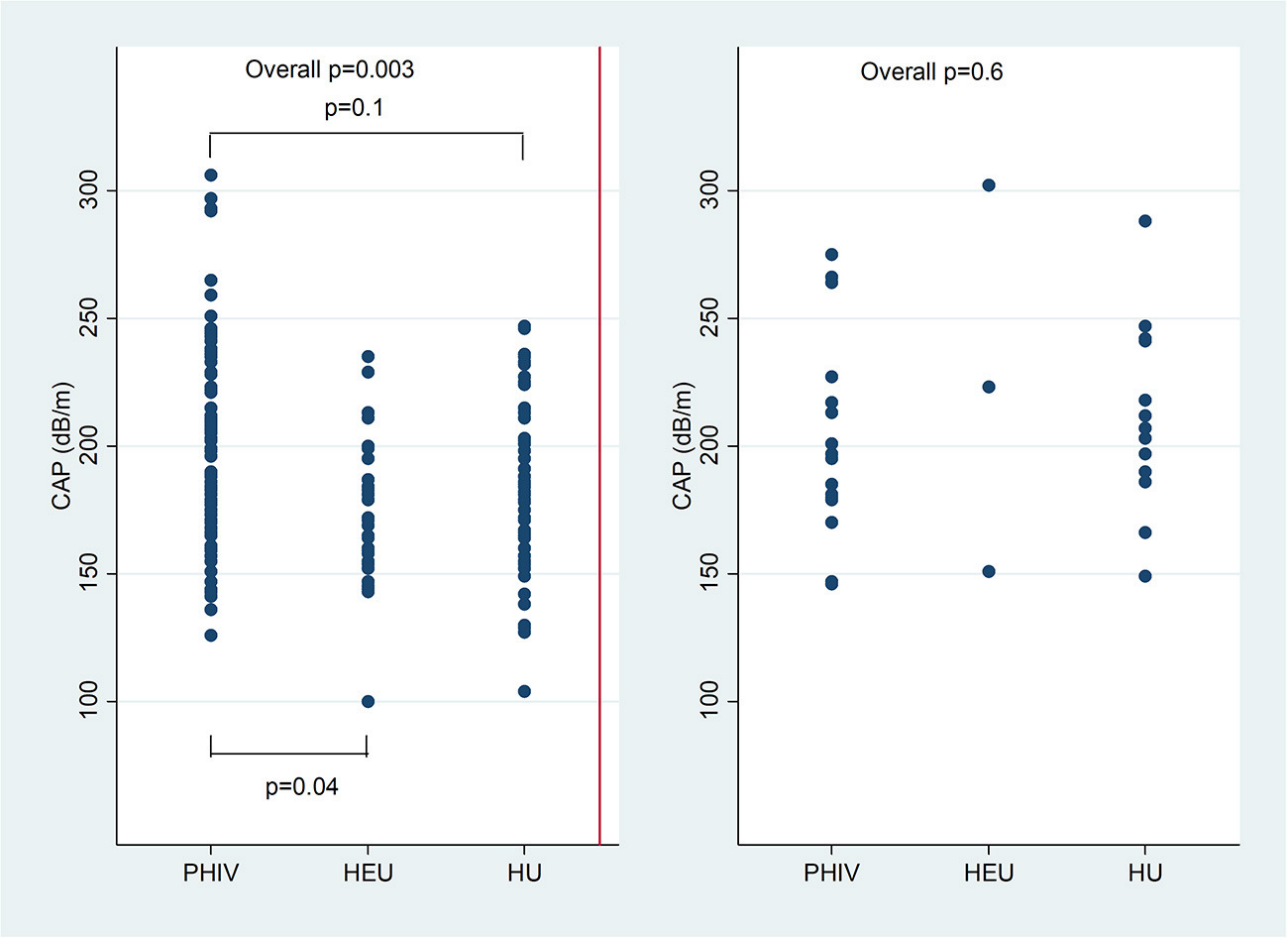
Controlled attenuation parameter (CAP) in dB/m comparing children with perinatally acquired HIV (PHIV), HEU and HU in lean children and overweight and obese children.

Overall CAP was higher in PHIV than HEU children (*p* = 0.004), but not HU children (*p* = 0.1). CAP was higher in lean PHIV children compared to both lean HEU (*p* = 0.002) and lean HU children (*p* = 0.02). There was no difference in CAP between overweight or obese PHIV, HEU and HU children (Figure 1).

**Figure 2:**
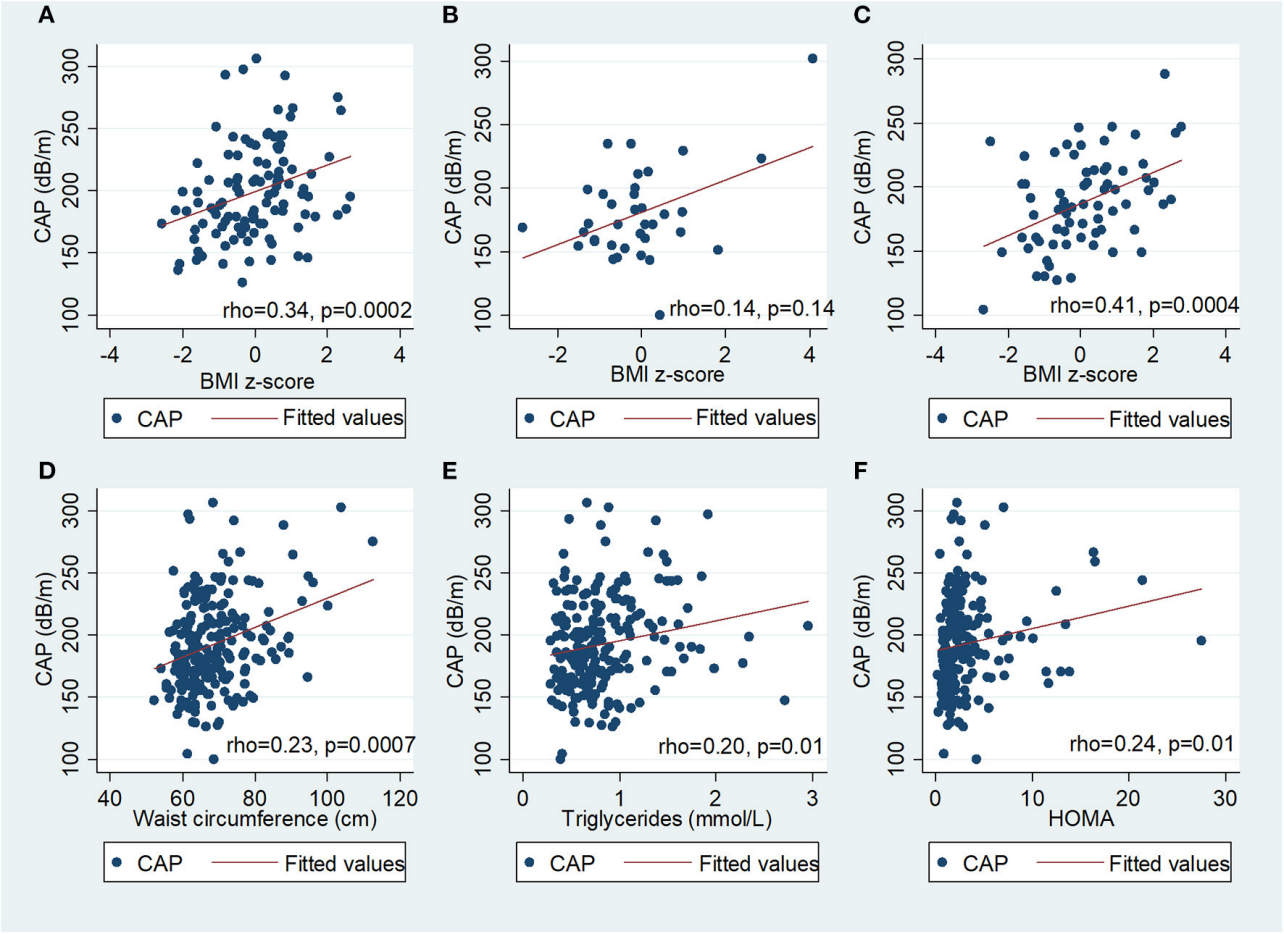
Scatter diagrams of controlled attenuation parameter (CAP) plotted against BMI z-score in **(A)** children with perinatally acquired HIV (PHIV), **(B)** HIV-exposed uninfected (HEU), **(C)** HIV-unexposed and against **(D)** waist circumference, **(E)** triglycerides and **(F)** HOMA in all children.

**Figure 3:**
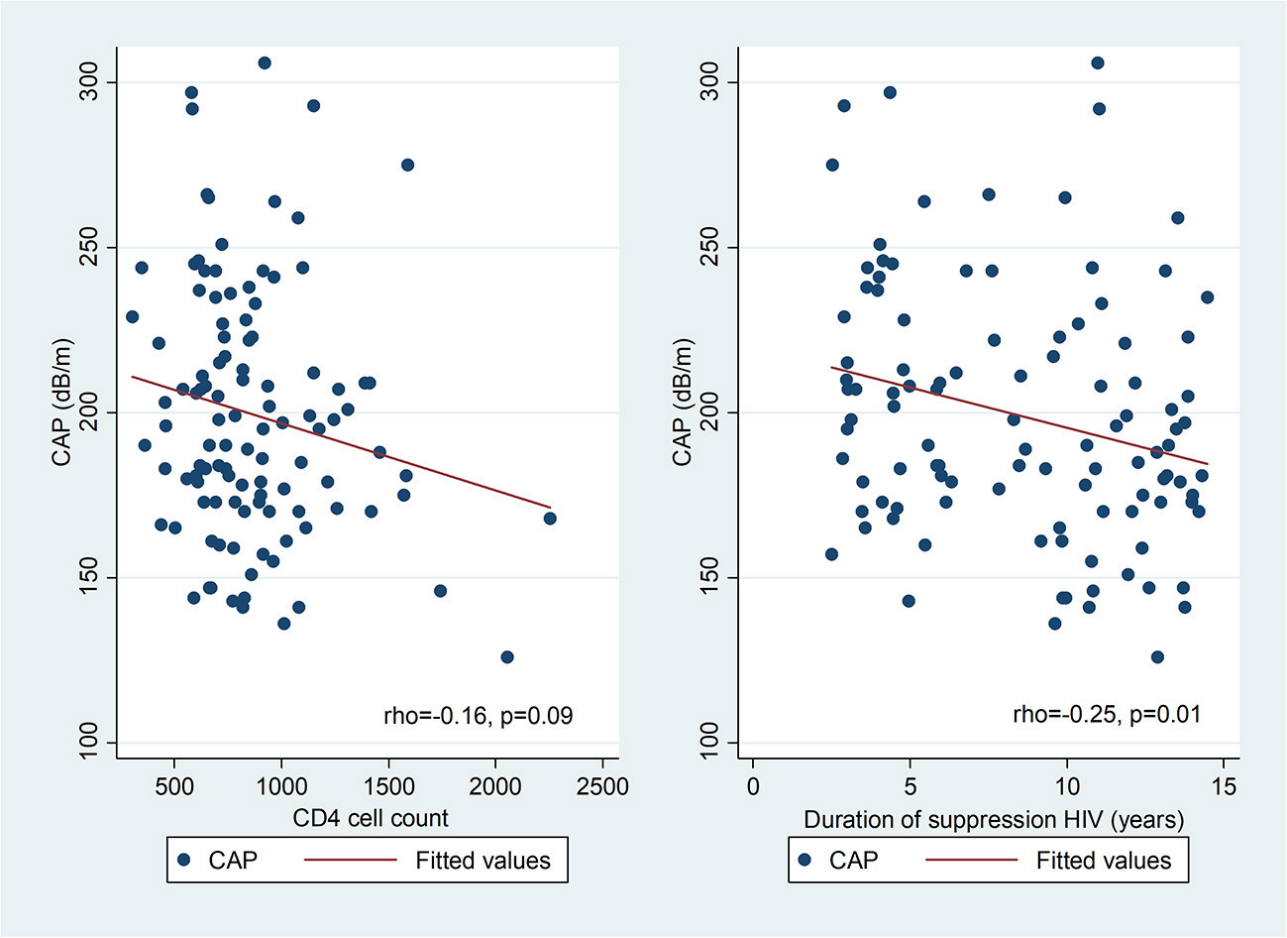
Scatter diagrams of controlled attenuation parameter (CAP) plotted against CD4 count and duration of suppression of HIV viraemia in PHIV.

In univariate analysis (Figure 2), there was a positive correlation between CAP and BMI z-score in PHIV (rho 0.34; *p* = 0.0002) and HU (Spearman's rho 0.41; *p* = 0.0004), waist circumference (Spearman's rho 0.23; *p* = 0.0007), triglycerides (Spearman's rho 0.18; *p* = 0.01), insulin (Spearman's rho 0.18; *p* = 0.01) and HOMA (Spearman's rho 0.18; *p* = 0.01), but not glucose, total, HDL or LDL cholesterol. There was a weak positive correlation between BMI z-score and CAP in HEU that was not statistically significant (rho 0.14; *p* = 0.14) CAP did not differ between boys and girls, nor with ethnicity. The coefficient of variation for CAP was 26%. In PHIV there was a significant negative correlation between duration of suppression of HIV viraemia and CAP (Spearman's rho−0.25; *p* = 0.01) and a non-significant weak negative correlation between CD4 count and CAP (Spearman's rho−0.16; *p* = 0.09) as indicated in Figure 3.

Using a CAP cut-off of >248 dB/m, 4 (29%) obese (2 PHIV), 1 (5%) overweight (1 PHIV) and 7 (4%) lean children (all PHIV) had hepatic steatosis (Fisher's exact *p* = 0.007). Hepatic steatosis was present in 10 (9%) PHIV, 1 (3%) HEU and 1 (1%) HU children (*p* = 0.08). Among lean children, 7 (8%) PHIV and no HEU or HU children had hepatic steatosis (*p* = 0.03). Agreement between hepatic ultrasound and CAP in diagnosing hepatic steatosis was 92% using an ultrasound hepatic steatosis score of grade ≥2 and CAP>248 dB/m (kappa 0.32; *p* = 0.0007), and 59% using an ultrasound score of grade ≥1 and CAP>248 dB/m (kappa 0.13; *p* = 0.02).

Ten PHIV children [median age 13.7 years (IQR 12.8–14.4 years; 8 (80%) female] were diagnosed with hepatic steatosis. All were virologically suppressed at the time of assessment, with a median duration of suppression of 6.5 years (IQR 4.0–11.0 years) and median CD4 cell count was 821 (IQR 654–1077). Two (20%) children were obese, 1 (10%) was overweight and 7 (70%) were lean. Of the children who had hepatic steatosis 4 were on zidovudine + lamivudine + lopinavir/ritonavir, 2 on zidovudine + lamivudine + nevirapine, 2 on abacavir + lamivudine + lopinavir/ritonavir, 1 on abacavir + lamivudine + efavirenz, and 1 on dolutegravir + darunavir + ritonavir at the time of assessment. CAP was higher in children currently on zidovudine (median 193dB/m, IQR 176–219 dB/m) compared to children not on zidovudine (median 184dB/m, 160–213dB/m; *p* = 0.04) and lopinavir/ritonavir (median 196 dB/m, IQR 177–221 dB/m) versus those not on lopinavir/ritonavir (median 184dB/m, IQR 166–211dB/m; *p* = 0.03).

In PHIV children, BMI z-score, waist circumference, CD4 count, zidovudine, lopinavir/ritonavir and duration of suppression of HIV viraemia were significantly associated with CAP in univariable regression (Table 3). In multivariable linear regression analysis controlling for age, sex and ethnicity, a unit increase in the BMI z-score increased CAP by 6% (*p* = 0.001); CAP reduced by 1% for a 100 unit increase in CD4 (*p* = 0.02) and each additional year of HIV suppression (*p* = 0.009). Age, sex and ethnicity were retained in all multivariable models as possible confounders. In HEU children, BMI z-score, waist circumference and waist-hip ratio were positively associated with CAP in univariable linear regression analysis (Supplementary Table 1). In HU children, BMI z-score, waist circumference, triglycerides and HOMA were positively associated with CAP (Supplementary Table 2). In multivariable linear regression analyses, CAP was positively associated with BMI z-score in HEU; and both BMI z-score and HOMA in HU (Supplementary Tables 1, 2).

**Table 3:** Univariable and multivariable linear regression analyses for predictors of controlled attenuation parameter (CAP) in PHIV children (*n* = 105).

	**Univariable**		**Multivariable**		
	**Coefficient**	***P*** **-value**	**Coefficient**	***P*** **-value**	**VIF**
Age (years)	+2%	0.1	−0.5%	0.7	1.14
Sex (male)	+5%	0.2	+0.2%	1.0	1.16
Ethnicity (African)	−8%	0.2	−2%	0.7	1.09
Tanner staging ≥ 2	−4%	0.6		-	
BMI z-score	+5%	0.001	+6%	0.001	1.22
Waist circumference (cm)	+5%	0.006		-	
Waist-hip ratio	+6%	0.9		-	
TG (mmol/L)	+5%	0.2		-	
Insulin (mIU/mL)	+0.04%	0.7		-	
HOMA	+0.2%	0.6		-	
ALT (u/L)	+0.3%	0.2		-	
CD4, per 100 cells/uL	−1%	0.04	−1%	0.02	1.02
Duration of HIV suppression (years)	−1%	0.008	−1%	0.009	1.22
Zidovudine	+6.1%	0.03			
Lopinavir/ritonavir	+6.6%	0.02			

*ALT, alanine transaminase; HOMA, homeostatic model assessment; PHIV, children with perinatally acquired HIV; TG, triglycerides; Coefficients represent % change in CAP per unit change in input variable; model R^2^ = 0.23*.

## Discussion

In this cohort of clinically well South African children, 9% of PHIV children on long-term suppressive ART had hepatic steatosis, compared to 3% in HEU and 1% in HU children. In multivariable linear regression analysis of PHIV controlling for age, sex and ethnicity, BMI z-score was positively associated with CAP, while CD4 count and duration of suppression of HIV viraemia were negatively associated with CAP. In our cohort, few children had evidence of hepatic fibrosis, only 2% of PHIV children assessed by APRI, none using FIB-4 and 7% using transient elastography.

A recent Spanish study of PHIV children, adolescents and youth on ART (86.8% suppressed) found a higher NAFLD prevalence of 28.9%, compared to 7.9% in controls, that was only partly explained by overweight and metabolic factors (34). A study of young American PHIV adults on ART (72% suppressed) found that despite a higher prevalence of hepatic steatosis than controls (33 vs. 10%), metabolic parameters such as waist circumference were risk factors rather than HIV-related factors. Also, transient elastography and APRI values were similar to controls (33). The median age of our cohort is younger than these cohorts both of which included adults. A lower prevalence of NAFLD has been described in African populations and this, in addition to the fact that PHIV children in our cohort started ART early in life and have remained on treatment with good clinical care, might also contribute to our observation.

In a study of Asian PHIV adolescents virologically suppressed on ART, the prevalence of hepatic steatosis using ultrasonography was 8% and hepatic fibrosis using TE was 9%, similar to the rates of hepatic steatosis and fibrosis using transient elastography with CAP in our study (35). In a French study from 2009, PHIV children had higher liver stiffness than HU controls with 17% having mild hepatic steatosis on ultrasound (36). In our study 1% of PHIV children had advanced fibrosis using TE, which is similar to that reported in previous studies of PHIV children, ranging from 0.8 to 3.2% (37–39). Using non-invasive markers such as APRI and FIB-4, liver fibrosis has been found in up to 8.5% of PHIV (37–39). Although non-invasive hepatic fibrosis scores developed for adults perform poorly in children, APRI has been found to have a fair diagnostic accuracy for detecting the presence of any fibrosis (12). Despite their limitations in children, APRI and FIB4 can be easily calculated from routinely available laboratory test results and have been used in previous studies investigating liver disease in PHIV children. In our study no children had any hepatic fibrosis using FIB4 and only 2% using APRI.

Unsuppressed HIV viraemia in adults is a risk factor for hepatic steatosis and fibrosis, while suppressive ART is protective (9, 40–42). There is also concern of potential long-term ART effects including dyslipidaemia, weight gain, mitochondrial toxicity and insulin resistance, with children facing several decades more of ART than adults (8, 43, 44). In our study both zidovudine and lopinavir/ritonavir use were associated with hepatic steatosis. Although Africa has the lowest reported prevalence of NAFLD worldwide at 14% and black ethnicity is considered protective against NAFLD, little data is available from African countries and it is likely the disease burden is underestimated (45–49). In liver biopsy studies performed in South African adults with HIV, hepatic steatosis was present in 19–21%; 28% of those with steatosis had steatohepatitis (50, 51).

There are concerns regarding the emergence of NCDs in individuals living with HIV. Many PHIV children in low-income settings have multisystem chronic comorbidities, but the NAFLD burden in PHIV in LMICs is not known (52, 53). Although it appears that few PHIV children with suppressed viremia have liver fibrosis, in sub-Saharan Africa there are 1.7 million children living with HIV, many of whom did not commence ART as infants and may not have sustained viral suppression. Thus, even a small proportion of African PHIV developing progressive liver fibrosis may lead to a significant disease burden at a population level.

This study provides a unique window into the future of the current global generation of early-treated PHIV children. Universal early ART for infants with PHIV has been standard-of-care for only just over a decade. Our cohort of PHIV children is the oldest homogenous cohort of early-treated African PHIV children, was largely spared the cumulative organ damage from repeated opportunistic infections, but have accumulated extensive ART exposures from early in life. To the best of our knowledge this is the largest study to evaluate PHIV children on suppressive ART from early life using transient elastography with CAP, in addition to routine non-invasive hepatic investigations. Children in this cohort were recruited from the same local communities and socio-economic background.

A limitation of this study is that liver ultrasounds could not be performed post-Covid lockdown. Testing for hepatitis B or C was only performed in children with an abnormality on non-invasive hepatic investigations. However, due to excellent vaccine coverage, the prevalence of hepatitis B in South African children is only 0.4% and hepatitis C prevalence in the South African adult population is 1.1%. In a large cohort of HIV-exposed South African children, none had hepatitis C infection (54–56). It is unlikely cases were missed as children in this cohort have liver enzymes measured annually and are routinely investigated for hepatitis B and C if any abnormality is detected. A further limitation is that our study was cross-sectional, with children requiring longitudinal follow-up to evaluate changes in hepatic steatosis or fibrosis over time. In addition, these children were recruited onto two clinical trials and followed over many years from birth or early life so they may not reflect the real life situation of PHIV children born at the same time who may have been diagnosed and treated later in life. Non-invasive imaging techniques such as TE and CAP have a lower sensitivity in diagnosing hepatic steatosis in the early stages, which might be greater in children as they have had fewer years for disease progression. This might also contribute to the low prevalence of hepatic steatosis observed in our study. A further limitation is that there were differences in ethnicity between the HU and PHIV and HEU children which might be an important confounder.

Recently released South African guidelines recommend using TE and CAP in those living with HIV on long-term ART (57). Who should be screened and how, has not yet been determined. The natural history of NAFLD in PHIV children is not known and it is unclear which children will develop NASH or hepatic fibrosis and over what period of time this will develop. There are limitations in the sensitivity of all non-invasive diagnostic modalities to screen for and diagnose NAFLD in children currently available, including ultrasound, transaminases and newer modalities such as transient elastography with controlled attenuation parameter. Although TE and CAP are relatively new and not as well established in children as in adults, we decided to use these tests as they have been used in children with NAFLD, the equipment was available to us and TE is rapid, non-invasive and well tolerated by children.

### Conclusions

The prevalence of hepatic steatosis was low, but higher in lean PHIV than lean HEU and HU controls evaluated by transient elastography with CAP. Very few PHIV, HEU and HU children had evidence of hepatic fibrosis. In South African PHIV children on ART from early life increasing BMI was associated with increasing CAP, while higher CD4 counts and longer duration of HIV suppression were associated with decreasing CAP although the association was mild. This suggests that in South African PHIV children longer duration of HIV suppression and higher CD4 count may be protective against hepatic steatosis, although being overweight and obese remain risk factors for hepatic steatosis. Measures to prevent excessive weight gain in all children regardless of HIV status together with good HIV management must be applied.

## Data Availability Statement

The raw data supporting the conclusions of this article will be made available by the authors, without undue reservation.

## Ethics Statement

The studies involving human participants were reviewed and approved by Stellenbosch University Health Research Ethics Committee. Written informed consent to participate in this study was provided by the participants' legal guardian/next of kin.

## Author Contributions

PR, EN, MC, RP, and SI designed the research study. PR and SI recruited participants to the study. PR and KO analysed the data. PR performed the research and the Fibroscans and wrote the original draft manuscript. RP reported on the ultrasounds. EN, SI, MC, RP, KO, and SB discussed, revised, and contributed to the final version of the manuscript. All authors have read and approved the final manuscript.

## Funding

The work reported herein was made possible through funding by the South African Medical Research Council through its Division of Research Capacity Development under the SAMRC Clinician Researcher Programme (PR). This work was supported in part by a Fogarty International Center HIV Research Training Program grant, National Institutes of Health, to the University of Pittsburgh and Stellenbosch University (D43TW010937) and the Harry Crossley Foundation (PR). The CHER trial was supported by US National Institute of Allergy and Infectious Diseases (NIAID; CIPRA network, Grant U19 AI53217); NIH grants (R01 HD099846, R01 DC015984, R01 HD071664); the Departments of Health of the Western Cape and Gauteng, South Africa; and GlaxoSmithKline/ViiV Healthcare. CHER follow up studies were supported by NIH grants (R01 HD099846, R01 DC015984, R01 HD071664, MH105134). P1060 and P1104s were supported by the IMPAACT Network, with funding provided by the National Institute of Allergy and Infectious Diseases of the National Institutes of Health [Award Numbers UM1AI068632 (IMPAACT LOC], UM1AI068616 (IMPAACT SDMC), and UM1AI106716 (IMPAACT LC)]. SI was supported by research grants from University of California San Diego Centre for AIDS Research (UCSD CFAR) (#P30-AI036214); Fogarty International Centre Clinical Research Fellowship (#R24-TW007988); Collaborative Initiative for Paediatric HIV Education and Research (CIPHER) (#158-INN); Eunice Kennedy Shriver National Institute of Child Health and Human Development (#1R01HD083042); University of Cape Town Clinical Trials Unit (#UM1AI069519); South African Medical Research Council (#47884); and South African National Research Foundation (#29276). SB was supported by Eunice Kennedy Shriver National Institute of Child Health and Human Development (R01HD083042) and UCSD CFAR (P30-AI036214).

## Author Disclaimer

The content hereof is the sole responsibility of the authors and does not necessarily represent the official views of the SAMRC.

## Conflict of Interest

The authors declare that the research was conducted in the absence of any commercial or financial relationships that could be construed as a potential conflict of interest.

## Publisher's Note

All claims expressed in this article are solely those of the authors and do not necessarily represent those of their affiliated organizations, or those of the publisher, the editors and the reviewers. Any product that may be evaluated in this article, or claim that may be made by its manufacturer, is not guaranteed or endorsed by the publisher.
