# Supplementary material for: Prevalence and Risk Factors for Hepatic Steatosis in Children With Perinatal HIV on Early Antiretroviral Therapy Compared to HIV-Exposed Uninfected and HIV-Unexposed Children
Source: Front Pediatr. 2022 Jun 9;10:893579. doi: 10.3389/fped.2022.893579 (PMC9218275; doi:10.3389/fped.2022.893579)
Supplement: Supplementary file 1 [file Table_1.DOCX]

Table 4. Univariable and multivariable linear regression analyses for predictors of logarithmic transformation of controlled attenuation parameter (CAP) in HEU children (n=36; model R^2^=0.19) Coefficients represent % change in CAP per unit change in input variable.

|  | Univariable | | Multivariable | | | |  | |  |  |  |
| --- | --- | --- | --- | --- | --- | --- | --- | --- | --- | --- | --- |
|  | Coefficient | P-value | | Coefficient | P-value | | VIF | | | | |
| Age (years) | -2% | 0.1 | | -1% | 0.4 | | 1.28 | | | | |
| Sex (male) | +5% | 0.2 | | -6% | 0.4 | | 1.29 | | | | |
| Ethnicity (African) | -2% | 0.8 | | -0.3% | 1.0 | | 1.12 | | | | |
| Tanner staging ≥2 | -10% | 0.2 | | - | - | |  | | | | |
| BMI z-score | +6% | 0.03 | | +6% | 0.048 | | 1.12 | | | | |
| Waist circumference (cm)  Waist-hip ratio | +0.8%  +6% | 0.02  0.02 | | -  - |  | -  - | |  | | |  |
| TG (mmol/L) | -5% | 0.6 | | - |  | - | |  | | |  |
| Insulin (μIU/mL) | +0.5% | 0.3 | | - |  | - | |  | | |  |
| HOMA | +2% | 0.3 | | - |  | - | |  | | |  |
| ALT (u/L) | +1% | 0.009 | | - |  | - | |  | | |  |

ALT, alanine transaminase; HEU, HIV-exposed uninfected; HOMA, homeostatic model assessment; TG, triglycerides.
