# Supplementary material for: Prevalence and Risk Factors for Hepatic Steatosis in Children With Perinatal HIV on Early Antiretroviral Therapy Compared to HIV-Exposed Uninfected and HIV-Unexposed Children
Source: Front Pediatr. 2022 Jun 9;10:893579. doi: 10.3389/fped.2022.893579 (PMC9218275; doi:10.3389/fped.2022.893579)
Supplement: Supplementary file 2 [file Table_2.DOCX]

Table 5. Univariable and multivariable linear regression analyses for predictors of logarithmic transformation of controlled attenuation parameter (CAP) in HU children (n=67; model R^2^=0.30) Coefficients represent % change in CAP per unit change in input variable.

|  | Univariable | | Multivariable | |  |
| --- | --- | --- | --- | --- | --- |
|  | Coefficient | P-value | Coefficient | P-value | VIF |
| Age (years) | -1% | 0.4 | -3% | 0.06 | 1.09 |
| Sex (male) | +6% | 0.2 | +0.5% | 0.9 | 1.07 |
| Ethnicity | +6% | 0.2 | +5% | 0.3 | 1.05 |
| Tanner staging ≥2 | +10% | 0.2 | - | - |  |
| BMI z-score | +7% | <0.0001 | +7% | <0.0001 | 1.05 |
| Waist circumference (cm)  Waist-hip ratio | +0.6%  -25% | 0.01  0.5 | -  - | -  - |  |
| TG (mmol/L) | +20% | 0.03 | - | - |  |
| Insulin (μIU/mL) | +0.6% | 0.05 | - | - |  |
| HOMA | +2% | 0.05 | +3% | 0.035 | 1.14 |
| ALT (u/L) | +0.3% | 0.6 | - | - |  |

ALT, alanine transaminase; HOMA, homeostatic model assessment; HUU, HIV-unexposed uninfected; TG, triglycerides.
